# Longitudinal Study of Cognitive and Emotional Alterations in Amyotrophic Lateral Sclerosis: Clinical and Imaging Data

**DOI:** 10.3389/fneur.2021.620198

**Published:** 2021-07-08

**Authors:** Soumia Benbrika, Franck Doidy, Laurence Carluer, Audrey Mondou, Alice Pélerin, Francis Eustache, Fausto Viader, Béatrice Desgranges

**Affiliations:** Normandie Univ, UNICAEN, PSL Université Paris, EPHE, INSERM, U1077, CHU de Caen, GIP Cyceron, Neuropsychologie et Imagerie de la Mémoire Humaine, Caen, France

**Keywords:** amyotrophic lateral sclerosis, cognition, emotional processing, psychological assessment, neural correlate

## Abstract

**Objectives:** Extra-motor manifestations occur in 50% of patients with amyotrophic lateral sclerosis (ALS). These mainly concern cognition, emotional processing and behavior. Depression and anxiety are less frequent. Little is known about how these manifestations change as the disease progresses. Similarly, although cortical thinning has been well-documented at disease onset, there are scant data about cortical thinning over time and how this correlates with extra-motor manifestations. The present study therefore assessed cognitive, emotional and psychological state and cortical thinning in a group of patients with ALS at baseline and after a follow-up period.

**Methods:** We assessed executive functions, facial emotion recognition, depressive and anxious symptoms, and cortical thinning in 43 patients with ALS at baseline, comparing them with 28 healthy controls, and 21 of them 9 months later. We looked for links among the extra-motor manifestations and correlations with cortical thickness.

**Results:** At baseline, patients had poor executive function and recognition of complex emotions from the eyes, and more anxious and depressive symptoms than controls. At follow-up, only inhibition abilities had worsened. Cortical thinning was observed in bilateral pre-central regions and other parts of the cerebral cortex at baseline. Over time, it worsened in motor and extra-motor areas. Executive functions correlated with thinning in the middle and inferior frontal gyrus and orbitofrontal cortex.

**Conclusions:** During follow-up, there was little deterioration in extra-motor manifestations and psychological state, despite continuing cortical thinning. Patients with affective Theory of Mind (ToM) changes seemed less depressed than the others. Impaired mental flexibility was subtended by prefrontal regions with cortical thinning.

## Introduction

Amyotrophic lateral sclerosis (ALS) is a motor neuron disease. However, the brain lesions often extend beyond the primary motor areas, resulting in cognitive impairment and/or behavioral changes in ~50% of cases (1). The main cognitive impairment concerns executive functions (2, 3). Around 30% of patients have behavioral changes characterized by apathy and irritability (4). Facial emotion recognition, judgments of emotional valence, and social cognition skills such as decision-making and theory of mind (ToM) may also be impaired (5, 6). Most longitudinal studies assessing cognitive functions in ALS have reported an absence of change in this domain over time (3).

The prevalence of depression in ALS varies considerably (7) depending on the timing and the tools used for the investigation. In a study of 964 patients, 5%, were diagnosed with severe depression, 14% with moderately severe depression, and 33% with moderate depression (8). Anxiety occurs in 20-35% of patients. Despite the physical worsening, anxiety, and depression do not seem to increase over time.

Cerebral gray-matter changes have been studied by measuring either volume [e.g., (9)] or cortical thickness (CTH), which allows anatomical lesions to be picked up earlier (9). At disease onset, CTH is reduced in the pre-central gyrus (10, 11) and supplementary motor area (11) as well as in various extra-motor regions (10, 12, 13). Cognitively impaired patients with ALS (ALS-ci) have greater thinning in the bilateral pre-central gyrus, insular and cingulate cortices, and frontotemporal lobes than cognitively normal patients (14, 15). Longitudinal studies have shown that thinning increases over time in extra-motor regions (16, 17) but this is not necessarily the case in motor areas (18, 19). The links between these clinical/behavioral and structural changes over time have not yet been assessed.

The present study was designed to assess executive functions, emotional perception, psychological state, and cerebral cortical thickness in a group of patients with ALS at baseline, comparing them with healthy controls, and after a follow-up period. We also looked for links among the extra-motor manifestations and correlations between these manifestations and cortical thinning.

## Materials and Methods

### Participants

We recruited 43 patients (25 men and 18 women) with probable or definite ALS and 28 healthy controls matched for age, sex, and years of education. All participants had to be native French speakers aged more than 18 years, with more than 7 years of education. No patient had a severe cognitive deficit, as measured on the Mattis Dementia Rating Scale (MDRS ≥ 127), or fulfilled the criteria for a diagnosis of frontotemporal dementia (FTD) or primary progressive aphasia. Controls had good overall cognitive functioning (MDRS > 130).

All patients and controls were examined at baseline, and 21 patients were examined again 9–12 months later. Patients were rated on the ALS Functional Rating Scale-revised form (ALSFRS-R), the Norris ALS Bulbar Scale, and the Medical Research Council (MRC) Muscle Strength Scale. The MRC muscle scale grades muscle strength from 0 (no contraction) to 5 (normal functioning). In this study, we used it in 12 muscle groups: 6 in each upper limb (index abduction, wrist flexion and extension, elbow flexion and extension and shoulder flexion), 5 in each lower limb (hip flexion and abduction, knee flexion and extension, ankle flexion) and one in the neck (flexion and extension. The total ranges from 0 to 120 (20). At baseline, all were able to speak and/or write intelligibly and had a forced vital capacity above 50% of the predicted value. They gave their written informed consent, and the study was approved by the independent regional ethics committee.

### Psychological and Cognitive Assessment

Depressive symptoms were measured with the Beck Depression Inventory (BDI), and anxiety with the Spielberger State-Trait Anxiety Inventory (STAI-Y): STAI-Y 1 for state anxiety and STAI-Y 2 for trait anxiety.

In the cognitive assessment, global cognition was measured with the MDRS. Executive functions were assessed using the Hayling Sentence Completion Test (HSCT), Letter-Number Sequencing task (LN sequencing), Trail Making Test (TMT), and a letter and categorical verbal fluency task (Letter VF vs. Animal VF). Patients' verbal fluency performances were expressed as indices, as recommended by Abrahams et al. (21). For the TMT, we subtracted the processing time for Part A from the processing time for Part B (TMT B-A time). Patients were classified as having executive cognitive impairment when they failed at least two executive tests (as in Montuschi et al.) (22).

Facial emotion recognition was evaluated with the Face Eyes Test (FET), a French adaptation of the Reading the Mind in the Eyes Test (23). It consists of 40 black-and-white photographs of either the eye region (FET-eyes) or the whole face (FET-face) representing different facial expressions. Participants had to decode the expressions (basic or complex emotions) and select the appropriate emotion from three options (one target and two foils). Performances were expressed as four scores: FET-eyes basic emotions (FET-eyes-be), FET-eyes complex emotions (FET-eyes-ce), FET-face basic emotions (FET-face-be), FET-face complex emotions (FET-face-ce).

### Imaging

High-resolution T1-weighted anatomical images were acquired on an Achieva 3T scanner (Philips, Eindhoven, The Netherlands) using a three-dimensional fast field echo sequence. Cortical thickness measures were performed with the FreeSurfer image analysis suite (http://surfer.nmr.mgh.harvard.edu/) (24). To extract reliable longitudinal cortical thickness data, images were automatically processed with the longitudinal stream in FreeSurfer (25).

### Statistical Analyses

#### Clinical, Psychological, and Cognitive Data

Statistical analyses of demographic and clinical characteristics, as well as cognitive, psychological and facial emotion recognition data, were performed using STATISTICA 10.0 (StatSoft, Tulsa, OK, USA). The threshold of significance was set at *p* = 0.05. As some variables were not normally distributed, non-parametric tests were used.

At baseline, the Mann-Whitney *U* test was employed to compare patients and healthy controls on age, years of education, MDRS score, TMT B-A time, verbal and categorical fluency index, HSCT, LN sequencing, BDI, STAI-Y and FET scores. A chi-square test allowed us to compare the sex ratios.

At follow-up, we only considered patients who had been evaluated at both times. Their neurological, cognitive, psychological and emotion recognition scores were compared between baseline and follow-up using the Wilcoxon test.

Spearman's test was used to search for correlations between scores.

*Z* scores were calculated for executive functions, allowing us to identify executively impaired patients (22), referred to hereafter as *cognitively impaired patients*.

#### Imaging Analysis and Correlations

The statistical analysis was performed using Qdec, a FreeSurfer module developed to design and execute surface analysis. A Gaussian filter of 10 mm FWHM was used for smoothing in all analyses. Age at baseline was included in the model as a nuisance factor. To correct for multiple comparisons, we performed Monte-Carlo cluster-based simulation with 10,000 permutations. Clusters were considered significant at *p* < 0.05.

We ran a baseline group comparison (ALS < control). We then used symmetrized percent change (SPC) to evaluate longitudinal cortical thinning. SPC was the rate of change in cortical thickness between the two time points: [(thickness at follow-up – thickness at baseline)/follow-up time – baseline time]/[0.5 × (thickness at follow-up + thickness at baseline)]. The same process was used for the longitudinal analysis of abnormal clinical scores.

Finally, correlations were calculated between (1) abnormal clinical scores and CTH at baseline; (2) SPC of CTH and SPC of abnormal clinical scores at follow-up.

Results were visualized by overlaying significant cortical areas on inflated cortical surfaces. Figures were generated using pial surfaces.

## Results

### Clinical, Psychological, Cognitive, and Emotional Data

#### At Baseline

The demographic and clinical characteristics of patients and controls are set out in Table 1. ALSFRS-R scores ranged from 27 to 48, Norris scores from 16 to 39, and MRC strength scores from 63 to 120. Owing to fatigue, not all patients completed the cognitive/psychological work-up.

**Table 1:** Sociodemographic, clinical, psychological and cognitive data of patients and controls at baseline.

	**Patients**	**Controls**	***p*-value**
	**(*n* = 43)**	**(*n* = 28)**	
Sex (male/female)	25/18	17/11	0.05
Age (years)	61 ± 11	57.8 ± 8.9	0.13
Education level (years)	9.9 ± 2.9	11 ± 2.7	0.08
ALSFRS (/48)	38 ± 5.3	/	/
MRC (/120)	102 ± 15.3	/	/
Norris scale (/39)	34.6 ± 6.15	/	/
BDI (/39) (*n* = 33)	4.8 ± 4.49	1.9 ± 2.1	<0.001
STAI-Y 1 (/80) (*n* = 33)	34.9 ± 1.9	26.5 ± 6.13	<0.001
STAI-Y 2 (/80) (*n* = 33)	36.12 ± 10.2	34.5 ± 8.26	0.57
TMT B-A time (s) (*n* = 37)	67.9 ± 60.17	36.8 ± 31.3	0.01
Animal VF Index (*n* = 36)	3.7 ± 1.0	3.2 ± 0.9	0.08
Letter VF Index (*n* = 36)	7.78 ± 5.8	4.4 ± 1.22	<0.001
HSCT (*n* = 33)	5.3 ± 2.7	8.5 ± 3.2	<0.001
LN sequencing (*n* = 31)	8.7 ± 3.6	10.1 ± 2.64	0.07
FET-eyes be (*n* = 43)	5.95 ± 1.2	6.5 ± 1.5	0.06
FET-eyes ce (*n* = 43)	5.02 ± 1.6	5.8 ± 2.3	<0.001
FET-face be (*n* = 43)	7.06 ± 1.5	7.5 ± 1.6	0.22
FET-face ce (*n* = 43)	5.5 ± 1.7	7.1 ± 2.3	<0.001

*Values are mean ± SD. ALSFRS, ALS Functional Rating Scale; MRC, Medical Research Council Muscle Strength Scale; BDI, Beck Depression Inventory; STAI-Y, Spielberger State and Trait Anxiety Inventory; 1, State; 2, Trait; TMT, Trail Making Test; VF, Verbal Fluency; HSCT, Hayling Sentence Completion Test; LN sequencing, Letter-Number Sequencing task; FET, Face Eyes Test; be, basis emotions; ce, complex emotions. Significance set at p < 0.05*.

Patients scored higher than controls on both the BDI and STAI-Y (state but not trait anxiety) (Table 1). Patients scored lower than controls on the MDRS (*M*: 136 vs. 142; *SD*: 5.6 vs. 1.8; *p* < 0.001), TMT B-A, Letter VF and HSCT, but not on Animal VF or LN sequencing. Patients performed significantly more poorly than controls on FET-eyes-ce and FET-face-ce (Table 1). Overall, 40% of patients were cognitively impaired (i.e., impaired on at least two cognitive tests).

The only significant correlations concerned the FET-eyes-ce scores, which were negatively correlated with TMT B-A time (*r* = −0.4, *p* < 0.01) and the Letter VF index (*r* = −0.38, *p* = 0.03), and positively correlated with BDI scores (*r* = 0.4, *p* = 0.04).

#### At Follow-Up

A total of 21 patients underwent a follow-up examination after a mean duration of 9.28 months. The other 22 had either died in the meantime, been lost to follow-up, declined the second examination, or could not be assessed because of neurological worsening. The patients who reached the second examination had higher ALSFRS scores at baseline than those who did not. However, they did not differ on sociodemographic, cognitive, emotional and psychiatric parameters. The ratio of bulbar vs. spinal onset patients was not significantly different between the two groups (Table 2).

**Table 2:** Comparison of baseline sociodemographic, neurological, cognitive and emotional characteristics between patients included and not included at follow-up.

	**Patients not included at follow-up (*n* = 22)**	**Patients included at follow-up** ** (*n* = 21)**	***p* value**
Age (years)	62.5 ± 10.5	59.4 ± 11.6	0.40
Sex (male/female)	12/9	13/9	0.02
Education level (in years)	9.5 ± 3.0	10.3 ± 2.7	0.23
ALSFRS	35.8 ± 5.2	40.1 ± 4.6	<0.01
Norris scale	34.1 ± 5.8	35.1 ± 6.5	0.26
MRC	101.9 ± 13.7	102.3 ± 17.1	0.65
Spinal vs. Bulbar	16/6	16/5	0.07
BDI (/39)	3.3 ± 2.6	6.6 ± 5.6	0.06
STAI-Y 1 (/80)	34.2 ± 10.7	35.7 ± 11.7	0.64
STAI-Y 2 (/80)	34.8 ± 9.6	37.7 ± 6.1	0.56
TMT B-A time (s)	62.0 ± 58.1	74.9 ± 63.6	0.90
Animal VF Index	3.6 ± 1.1	3.9 ± 1.0	0.13
Letter VF Index	6.6 ± 2.2	9.2 ± 8.3	0.23
HSCT	5.0 ± 2.6	5.8 ± 3.0	0.40
LN sequencing	9.0 ± 3.9	8.3 ± 3.8	0.67
FET-eyes be	6.0 ± 1.3	6.1 ± 1.é	0.85
FET-eyes ce	5.0 ± 1.7	5.1 ± 1.7	0.85
FET-face be	7.0 ± 1.4	6.9 ± 1.9	0.87
FET-face ce	5.4 ± 1.6	5.7 ± 1.8	0.53

*Values are mean ± SD. ALSFRS, ALS Functional Rating Scale; MRC, Medical Research Council Muscle Strength Scale; BDI, Beck Depression Inventory; STAI-Y, Spielberger State and Trait Anxiety Inventory; 1, State; 2, Trait; TMT, Trail Making Test; VF, Verbal Fluency; HSCT, Hayling Sentence Completion Test; LN sequencing, Letter-Number Sequencing task; FET, Face Eyes Test; be, basis emotions; ce, complex emotions. MDRS, Mattis Demencia Rating Scale; Significance set at p < 0.05*.

Patients had lower ALSFRS-R, Norris and MRC strength scores than at baseline (Table 3). There were no changes in their BDI and STAI-Y scores. Patients had significantly lower cognitive performances, but only on the HSCT. FET performances were substantially the same. Overall, only four (20%) patients were cognitively impaired. All four were already cognitively impaired at baseline.

**Table 3:** Changes in neurological, psychological and cognitive scores of patients with ALS evaluated at baseline and follow-up.

	**Patients T1**	**Patients T2**	***p*-value**
ALSFRS (*n* = 17)	40.1 ± 4.6	29.7 ± 1.6	<0.001
Norris scale (*n* = 17)	35.1 ± 6.5	29.1 ± 12.4	<0.001
MRC (*n* = 17)	102.3 ± 17.1	81.2 ± 28.8	<0.001
BDI (/39) (*n* = 17)	3.3 ± 2.6	4.0 ± 4.5	0.5
STAI-Y 1 (/80) (*n* = 17)	34.2 ± 10.5	31.52 ± 9.7	0.1
STAI-Y 2 (/80) (*n* = 17)	34.8 ± 9.64	37.6 ± 11	0.4
TMT B-A (s) (*n* = 12)	62.0 ± 58.1	53.75 ± 38.1	0.9
Animal VF index (*n* = 16)	3.6 ± 1.1	3.7 ± 1.3	0.8
Letter VF index (*n* = 16)	6.6 ± 2.2	6.1 ± 2.0	0.7
HSCT (*n* = 16)	9.7 ± 2.4	5.2 ± 2.9	0.008
LN-sequencing (*n* = 10)	9.0 ± 3.9	9.6 ± 4.2	0.7
FET-eyes be (*n* = 21)	6.0 ± 1.2	6.0 ± 1.8	0.9
FET-eyes ce (*n* = 21)	4.8 ± 1.6	5.0 ± 1.8	0.7
FET-face be (*n* = 21)	7.0 ± 1.4	6.9 ± 1.8	0.9
FET-face ce (*n* = 21)	5.1 ± 1.6	4.9 ± 2.0	0.7

*Values are means ± SD. ALSFRS, ALS Functional Rating Scale; MRC, Medical Research Council Muscle Strength Scale; BDI, Beck Depression Inventory; STAI-Y, Spielberger State and Trait Anxiety Inventory; 1, State; 2, Trait; TMT, Trail Making Test; VF, Verbal Fluency; HSCT, Hayling Sentence Completion Test; LN sequencing, Letter-Number Sequencing task; FET, Face Eyes Test; be, basis emotions; ce, complex emotions. Significance set at p < 0.05*.

We only found two correlations, both negative: (1) between the Norris and STAI-Y (trait) scores (*r* = −0.6, *p* = 0.02), meaning that the more bulbar symptoms patients had, the more anxious they were, and (2) between the ALSFRS-R score and the Animal VF index (*r* = −0.4, *p* = 0.04), meaning that the more neurologically affected patients were, the poorer their verbal fluency performances.

### Cortical Thickness

#### At Baseline

**Figure 1:**
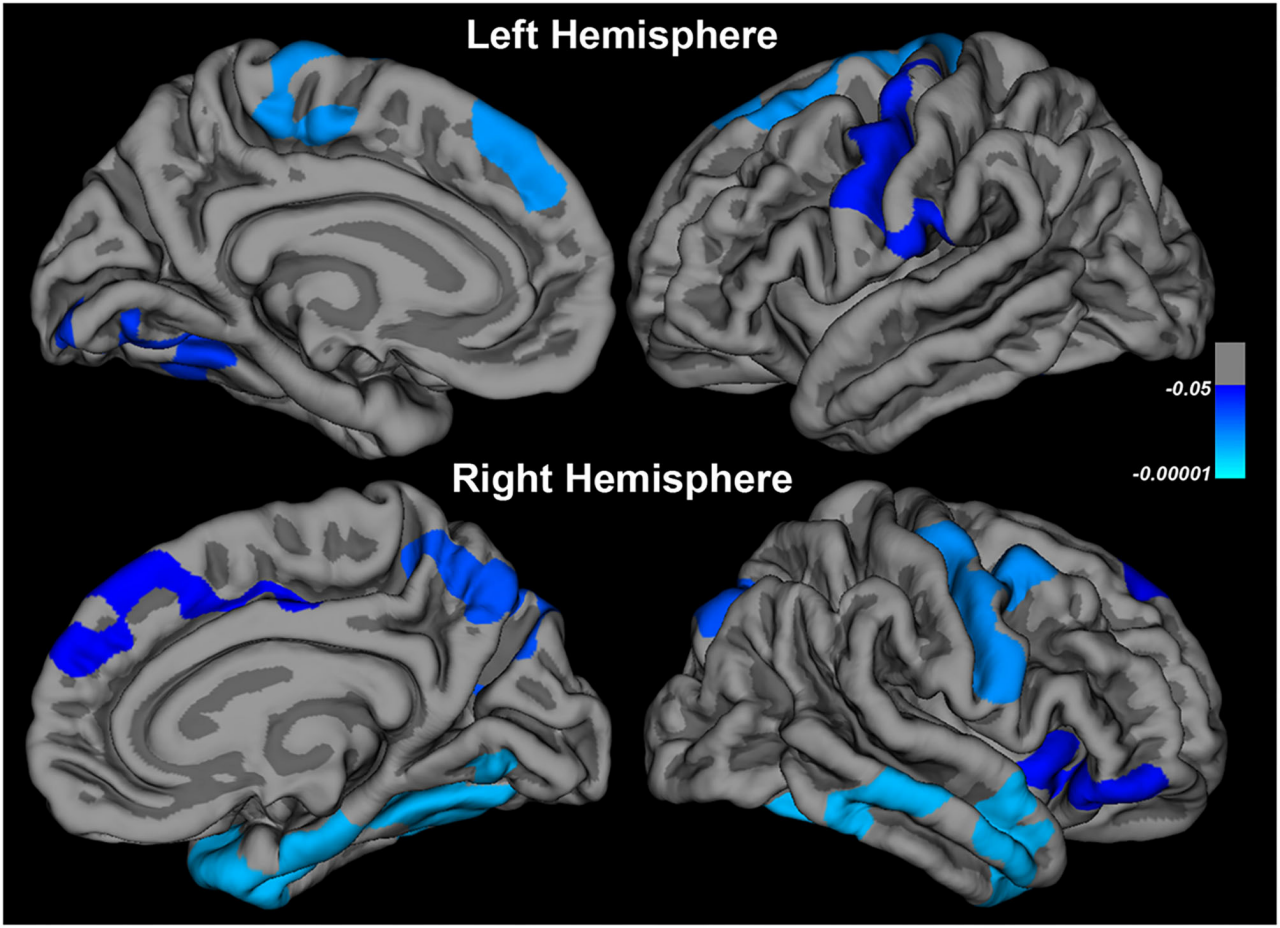
Changes in cortical thickness in patients at baseline compared to controls.

Comparison between patients (*n* = 40) and controls (*n* = 31) revealed cortical thinning in the regions indicated in Table 4 and Figure 1.

**Table 4:** Changes in cortical thickness in patients at baseline (compared to controls) and at follow-up (compared to baseline).

**At baseline**	**At follow-up**
• Right and left pre-central and post-central • Left paracentral • Right caudal middle frontal • Right and left superior frontal • Right insula • Right orbitalis • Right middle and superior temporal • Right inferior temporal • Right and left fusiform • Right superoparietal and occipital areas • Right and left lingual • Right posterior cingulate, • Right cuneus and precuneus • Left supramarginal	• Left and right pre-central • Left paracentral • Right post-central • Right caudal middle frontal • Right rostral middle frontal • Right triangularis and opercularis • Right orbitalis • Left lateral orbitofrontal and medial orbitofrontal • Left superior temporal • Left middle temporal • Left and right inferior temporal • Left and right fusiform • Left and right isthmus cingulate • Left and right lingual • Left and right parahippocampal • Left and right precuneus

**Figure 2:**
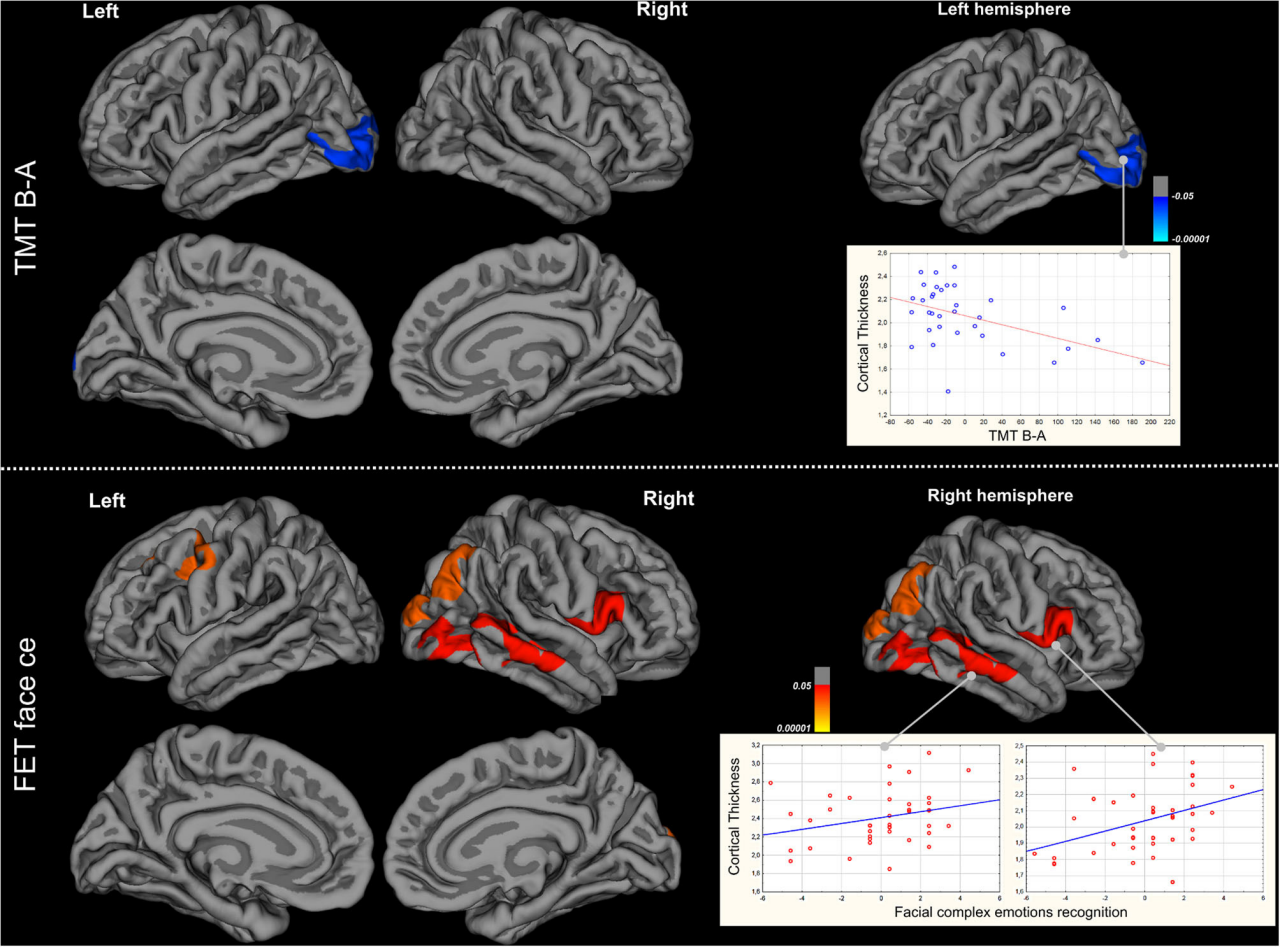
Correlations at baseline between cortical thickness and TMT B-A and recognition of complex facial emotions.

Significant positive correlations were found between scores on the FET-face-ce and CTH in the left caudal middle frontal, left superior frontal, left and rostral middle frontal, left and right pre-central, right pars opercularis and pars triangularis, right superior temporal, right inferior temporal, right lateral occipital and right inferior parietal cortices (*n* = 40; Figure 2). TMT B-A time correlated significantly and negatively with the CTH in left lateral occipital areas (*n* = 37; Figure 2). A part of CTH in the right middle temporal and left pre-central gyrus was both correlated with FET-face-ce performances and significantly reduced. No significant correlation was found between CTH and performances on VF, HSCT and LN-sequencing.

#### At Follow-Up

**Figure 3:**
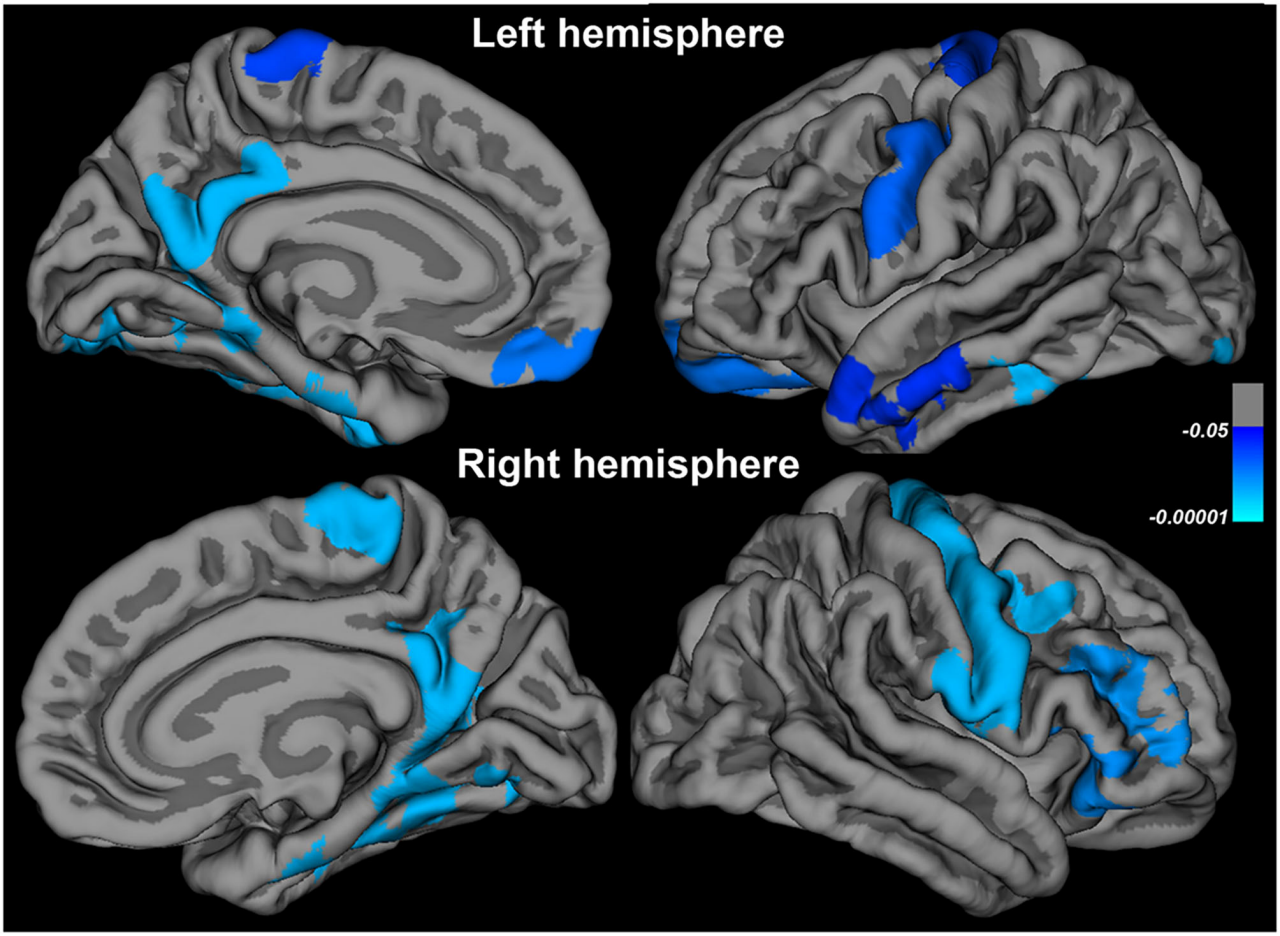
Changes in cortical thickness in patients at follow-up compared to baseline.

In the 17 patients, CTH significantly decreased between baseline and follow-up in the regions summarized in Table 4 and Figure 3.

**Figure 4:**
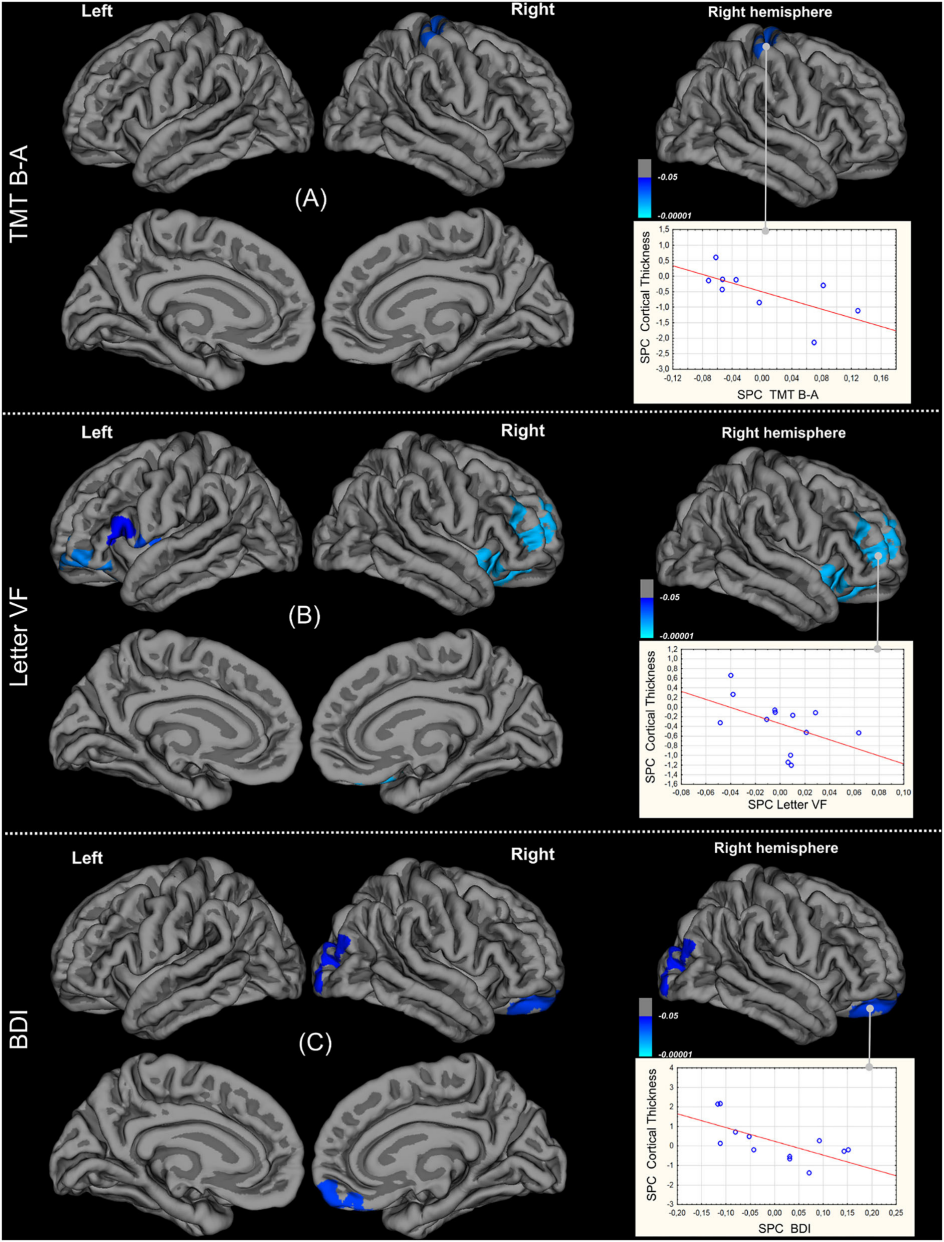
Correlations between changes of Cortical Thickness and those of abnormal clinical scores: TMT B-A **(A)**, letter-fluency index **(B)**, Beck Depression Inventory **(C)**.

In nine patients, significant negative correlations were found between TMT B-A time SPC and CTH SPC in the right pre- and post-central gyri, meaning that when cortical thinning increased in these regions, TMT B-A time increased (Figure 4A). CTH of concerned parts of pre and post-central gyri decreased significantly overtime. Significant negative correlation between Letter VF index and CTH SPCs are shown in Figure 4B (*n* = 13). When cortical thinning increased in the left pars opercularis and pars triangularis, left and right orbitalis, left and right insula, left and right lateral orbitofrontal and rostral middle frontal and left pre-central, right superior and middle orbitofrontal cortices, VF deteriorated. Furthermore, there was already significant cortical thinning in the right insula and left pars triangularis at baseline, and thinning increased over time in the right orbitalis, right lateral orbitofrontal and rostral middle frontal cortices. Significant negative correlations between BDI and CTH SPCs were found in the right lateral orbitofrontal, medial orbitofrontal and rostral middle frontal areas, such that the patients with more depressive symptoms (*n* = 12) had more cortical thinning in these regions (see Figure 4C). Finally, no significant correlation was found between CTH and Animal VF and HSCT SPCs.

## Discussion

### Cognitive Modifications in ALS

At baseline, nearly 40% of our patients presented an executive cognitive impairment, with the most affected executive functions being inhibition and set-shifting abilities. The decrease in phonemic but not semantic fluency fits in with an executive rather than a semantic dysfunction. Patients also had impaired recognition of complex (but not basic) emotions. Our results are similar to those of Andrews et al. (26) and Woolley and Rush (27) and suggest a deficit in affective ToM. Trojsi et al. (28) also reported a deterioration in scores on the Emotional Attribution Task (affective ToM) and emotional recognition, while Van der Hulst et al. (29) found that affective ToM was impaired in 36% of their patients with ALS. The significant correlations between complex emotion recognition performances and TMT B-A time and letter VF suggest that in our patients, affective ToM performances were influenced, at least in part, by executive dysfunction.

Cognitively impaired patients were under-represented at follow-up, compared to baseline. This can probably be attributed to a higher rate of attrition in patients with cognitive impairment at baseline owing to faster neurological decline, as suggested by the reported correlation between ALSFRS-R and Letter VF scores (30). All the patients without cognitive impairment at baseline remained preserved in our study. At the group level, in those who reached the follow-up examination, only HSCT scores were significantly lower, pointing to deterioration in inhibition abilities. Inhibition of action, i.e., behavioral inhibition, is thought to share the same neurophysiological bases as inhibition of attention. Among the behavioral changes reported in ALS, disinhibition has been shown to increase over time (3). Woolley et al. (31) found increased disinhibition overtime, that correlated to rapid disease progression and ALS-FRS scores. This change could be underpinned by deterioration of inhibition capacities as measured by appropriate cognitive tests. The longitudinal assessment of executive function has given contrasted results. In Elamin et al.'s study. (32), patients with initial cognitive deficits worsened. Similarly, Chiò et al. (33) found in a large sample of patients (including some with ALS-FTD) that the proportion of cognitively impaired participants increased across the clinical stages. On the other hand, Lulé et al. (34) suggested that the clinical trajectory might be predicted by the initial clinical phenotype, with a greater cognitive and behavioral deterioration in ALS associated to fronto-temporal dementia. This assumption is consistent with the fact that in our study, no patient cognitively intact at baseline developed a cognitive impairment at follow-up. Complex emotion recognition abilities did not change, which is partly in line with other studies. Gillingham et al. (35) found an improvement in the identification of angry faces, but not of other emotions, and performances on ToM tasks did not change. Trojsi et al. (36) found normal ToM at baseline in 21 patients with ALS, but patients with bulbar onset declined on both cognitive and affective ToM, whereas only cognitive ToM was impaired at follow-up in those with spinal onset.

### Psychological Adjustment in ALS

At baseline, 49% of our patients had mild to severe signs of depression, 22% had moderate to very high state anxiety, and 11% had moderate to high trait anxiety. Previous studies also using the BDI reported rates of 20-40% (37, 38) with a higher prevalence the years before and after diagnosis. We found no significant correlations between depression or anxiety and neurological status, as in most previous studies (39). By contrast, depressive symptoms correlated with eyes emotion recognition scores, such that the more depressed they were, the higher they scored on this affective ToM task. To our knowledge, no such correlation had previously been found. Individuals who have been diagnosed with major depressive disorder are known to exhibit an attentional bias toward negative emotional cues, with heightened vigilance and selective attention toward sad expressions and away from happy ones. When the ability to recognize facial (especially neutral and negative) emotions is preserved, patients are more likely to be depressed. This may mean that in ALS, the loss of affective ToM to some extent protects patients from the suffering that might be expected with such a dreadful disease.

At follow-up, we noted no significant changes in either BDI or STAI-Y scores, despite the neurological deterioration. This had already been observed (35, 37). Patients' psychological state may thus be primarily driven by the knowledge of the diagnosis rather than by its physical consequences, in terms of disability. Bulbar symptoms were positively correlated with anxiety, in line with some but not all studies (40).

### Cortical Thinning in ALS

At baseline, we observed cortical thinning in the right and left motor regions (pre-central, post-central, and paracentral), as well as in non-motor areas including the prefrontal cortex (superior and middle frontal, orbitalis), right insula, temporal areas (superior and middle temporal, fusiform) and small parts of the parietal and occipital lobes.

At follow-up, we observed further thinning in the right and left motor areas (pre-central, post-central, and paracentral), as well as in the prefrontal cortex, extending to the orbitofrontal cortex, and the right and left hippocampal and fusiform regions. Most longitudinal studies have reported similar results regarding cortical thinning in extra-motor areas (14, 18) but there is an ongoing debate about changes of CTH in motor areas over time (16).

### Neural Substrates of Extra-Motor Manifestations in ALS

At baseline, FET-eyes-ce scores were positively correlated with CTH in the left middle and inferior frontal gyri (caudal and rostral middle frontal, pars triangularis, pars opercularis), left and right premotor areas, right superior and inferior temporal cortices, and right parietal and occipital areas. FET-eyes-ce scores were also correlated with some cortical thinning in the left pre-central and middle temporal areas, suggesting that damage to these regions contributes to the affective ToM deficit in ALS. The role of the inferior frontal gyrus and orbitofrontal and middle frontal gyri in affective ToM is well-recognized, as a result of research on the consequences of damage in these areas, as well as functional MRI studies in healthy individuals. The superior temporal lobe and pre-central cortex are also involved in ToM. Our study is the first to report correlations between cortical thinning and affective ToM, and extends and reinforces results yielded by voxel-based morphometry (41) showing the involvement of the ventromedial and orbitofrontal cortices in affective ToM in ALS.

Taking into account the variations between baseline and follow-up, we also found correlations between (1) the decrease in verbal fluency and cortical thinning in the right and left middle and inferior frontal gyri, right and left orbitofrontal cortex, and right and left insula, and (2) the increase in TMT B-A time and cortical thinning in the pre- and post-central gyri. The cortical thickness of some of these regions was reduced at baseline and/or decreased between the two scans: right pre- and post-central, right orbitalis, right insula, right lateral orbitofrontal and rostral middle frontal gyri. This suggests that cortical thinning in these areas is implicated in the executive deficit in ALS. Consonni et al. (42) also reported a greater impairment of cortical thickness in the inferior frontal cortex and insula in patients with cognitive impairment, including executive deficit. The present study is one of the first to establish correlations between executive function and cortical thinning, and suggests that the decline in verbal fluency may be caused by impairment of the cortical circuit of mental flexibility.

Finally, we found a negative correlation between variations in depressive symptoms over time and variations in CTH in the right orbitofrontal cortex and middle frontal areas, such that the greater the cortical thinning in these regions, the more depressed the patients. Mood depressive disorder has been found to be associated with cortical and subcortical abnormalities, including structural and functional changes. The ventromedial prefrontal cortex is a core brain region for depression. Functional imaging shows that it is hyperactive during a depressive state, although its volume or thickness has been found to be either reduced or increased. It is therefore difficult to reach any firm conclusion about the role of the orbitofrontal cortex and middle frontal regions in the psychological state of patients with ALS.

The absence of correlation between neurological scores deterioration and cortical thinning could be due to a slow progression of the disease in our patients' sample. A more likely explanation is that the progression of muscle weakness depends on the involvement, not only of the central motor pathway, but also of spinal and/or brainstem neurons, that are beyond the reach of cerebral imaging.

Our study had some limitations. First, it would have been preferable to have a control group for the follow-up assessment, especially regarding changes in cortical thickness. This would have allowed us to distinguish between what was relevant to the disease itself and what was simply age-related. That said, changes in cortical thickness are unlikely to occur within the space of 9 months in the absence of a pathological process. Second, not all patients underwent all the tests, which sometimes reduced our sample size. We did not use tools specifically designed for use in ALS, but all the patients included in the present study were capable of taking the tests, and we took the impact of the illness on verbal fluency into account when calculating the indices. A limit of our study is that a significant number of patients were lost to follow-up. This may have influenced the results, particularly the respective proportions of cognitively impaired vs. unimpaired patients at the second assessment. However, even if patients who did not reach the follow-up examination were more neurologically impaired at baseline than those who did they did not differ on sociodemographic, cognitive, emotional and psychiatric parameters.

## Conclusion

Longitudinal studies of extramotor manifestations of ALS are scarce. Our work adds to the knowledge in this area by demonstrating that even in the case of an initial deficit in executive functions and affective ToM performances, there is little, if any, deterioration in these domains overtime -at least in patients with minimal impairment. Despite continuing physical deterioration, psychiatric symptoms do not worsen over time. It would appear that patients with affective ToM impairment are less depressed than the others. Then again, bulbar signs are a source of anxious manifestations. In parallel with this, cortical thinning continues, with involvement extending to extramotor regions, notably the prefrontal and temporal cortices. We also found that cortical thinning in some regions (middle and inferior frontal gyri and orbitofrontal cortex) seems to be responsible for reduced mental flexibility in ALS. Further studies are needed to understand the reasons for the different changes in motor and extramotor disorders.

## Data Availability Statement

The raw data supporting the conclusions of this article will be made available by the authors, without undue reservation.

## Ethics Statement

The studies involving human participants were reviewed and approved by CPP-Nord ouest no: 2008-A01150-55. The patients/participants provided their written informed consent to participate in this study.

## Author Contributions

SB was actively involved in this study from design to drafting. She conduced all the statistical analyses and played a central role in interpreting the results and writing the article. FD greatly contributed to the neuroimaging part of the study. LC, AM, and AP participated in the acquisition of clinical and cognitive data, especially in the careful screening of our cohort. FD, LC, and FE undertook the critical revision of the manuscript. BD and FV supervised and coordinated the teamwork from start to finish. Their knowledge and expertise in neuropsychology and ALS pathology were crucial for the design of the project and the analysis and interpretation of the results. They were also closely involved in revising the manuscript. All the authors read and approved the final manuscript.

## Conflict of Interest

The authors declare that the research was conducted in the absence of any commercial or financial relationships that could be construed as a potential conflict of interest.
